# Piericidin A Aggravates Tau Pathology in P301S Transgenic Mice

**DOI:** 10.1371/journal.pone.0113557

**Published:** 2014-12-01

**Authors:** Matthias Höllerhage, Roman Deck, Anderson De Andrade, Gesine Respondek, Hong Xu, Thomas W. Rösler, Mohamed Salama, Thomas Carlsson, Elizabeth S. Yamada, Seham A. Gad El Hak, Michel Goedert, Wolfgang H. Oertel, Günter U. Höglinger

**Affiliations:** 1 Dept. of Neurology, Philipps-Universität, Marburg, Germany; 2 German Center for Neurodegenerative Diseases, Dept. for Translational Neurodegeneration, Munich, Germany; 3 Department of Neurology, Technische Universität München, Munich, Germany; 4 Department of Toxicology, Mansoura University, Mansoura, Egypt; 5 Department of Pharmacology, Institute of Neuroscience and Physiology, Sahlgrenska Academy, University of Gothenburg, Gothenburg, Sweden; 6 Experimental Neuropathology Laboratory, Federal University of Pará, Belém, Brazil; 7 Division of Neurobiology, University of Cambridge, Cambridge, United Kingdom; Centre Hospitalier de l'Université Laval, Canada

## Abstract

**Objective:**

The P301S mutation in exon 10 of the tau gene causes a hereditary tauopathy. While mitochondrial complex I inhibition has been linked to sporadic tauopathies. Piericidin A is a prototypical member of the group of the piericidins, a class of biologically active natural complex I inhibitors, isolated from *streptomyces spp.* with global distribution in marine and agricultural habitats. The aim of this study was to determine whether there is a pathogenic interaction of the environmental toxin piericidin A and the P301S mutation.

**Methods:**

Transgenic mice expressing human tau with the P301S-mutation (P301S^+/+^) and wild-type mice at 12 weeks of age were treated subcutaneously with vehicle (N = 10 P301S^+/+^, N = 7 wild-type) or piericidin A (N = 9 P301S^+/+^, N = 9 wild-type mice) at a dose of 0.5 mg/kg/d for a period of 28 days via osmotic minipumps. Tau pathology was measured by stereological counts of cells immunoreative with antibodies against phosphorylated tau (AD2, AT8, AT180, and AT100) and corresponding Western blot analysis.

**Results:**

Piericidin A significantly increased the number of phospho-tau immunoreactive cells in the cerebral cortex in P301S^+/+^ mice, but only to a variable and mild extent in wild-type mice. Furthermore, piericidin A led to increased levels of pathologically phosphorylated tau only in P301S^+/+^ mice. While we observed no apparent cell loss in the frontal cortex, the synaptic density was reduced by piericidin A treatment in P301S^+/+^ mice.

**Discussion:**

This study shows that exposure to piericidin A aggravates the course of genetically determined tau pathology, providing experimental support for the concept of gene-environment interaction in the etiology of tauopathies.

## Introduction

Tau is a predominantly neuronal protein of which six major isoforms are generated by alternative splicing [1]–[4] from one gene *MAPT*, localized on the long arm of chromosome 17 (17q21) [5]. Tau is involved in the assembly and stabilization of microtubules [6]. Thus it plays an important role in axonal transport and neuronal viability. Intracellular aggregation of hyperphosphorylated tau protein is the histopathological hallmark of a group of neurodegenerative diseases, called tauopathies [7], [8]. The pathological hyperphosphorylation of tau-protein is considered as a possible early step in the formation of tau-aggregates in tauopathies [9].

The etiology of neurodegenerative tauopathies is multifactorial. Monogenetic *MAPT* mutations are responsible for some hereditary tauopathies, where so far, 51 disease-causing *MAPT* mutations are known [4]. One of these is the P301S mutation in exon 10, which leads to a substitution of the proline at position 301 by serine. The P301S mutation was first described in 1999 in families showing symptoms of corticobasal degeneration and frontotemporal dementia [10], [11]. Further work demonstrated, that there are two predominant clinical phenotypes in the patients carrying this mutation. Some show mainly parkinsonism similar to patients with progressive supranuclear palsy (PSP), while others show mainly symptoms of frontotemporal dementia [12]. This observation strongly suggests that independent genetic or environmental factors appear to shape the clinical phenotype of the disease caused by the P301S *MAPT* mutation.

In contrast to the purely genetically caused tauopathies described above, there are other tauopathies that appear to originate from exposure to a specific environmental factor. One prototypic example is the atypical Parkinson syndrome with tau pathology on the Caribbean island of Guadeloupe. Epidemiological studies have linked the disease to a high consumption of products from Annonaceae plants [13]–[15]. These plants contain high amounts of acetogenins, a class of lipophilic and potent inhibitors of complex I of the mitochondrial respiratory chain [16], [17]. The major representative of the annonaceous acetogenins is annonacin [18]. Systemic exposure to annonacin for 28 days induced neurodegeneration in rats *in vivo* [19]. Treatment of cultured striatal neurons from embryonic rats led to the accumulation of hyperphosphorylated tau in the cell soma of these cells [20]. These data strongly suggest that an environmental factor can trigger a tauopathy.

Between these two extremes of tauopathies, with solely monogenetic or environmental causes, the vast majority of sporadic cases are considered to result from an interaction of genetic predispositions (e.g. [21]) and environmental triggers or modifiers.

However, not much is known about environmental factors that could either trigger or modify the course of a genetically determined tauopathy. Since the consumption of products from Annonaceae plants is uncommon in North America or Europe, the question arises, which other environmental factors could be relevant in the etiology of tauopathies. A previous *in vitro* study showed, that a broad spectrum of natural complex I inhibitors can induce tau pathology and cell death in cultured neurons [22]. One of the most potent natural neurotoxins to induce somatodendritic accumulation of phosphorylated tau and cell death in nanomolar concentrations is piericidin A [22]. Piericidin A, is the most common member of the family of piericidins, a class of potent complex I inhibitors synthesized by *streptomyces spp.* [23], [24]. These bacteria are ubiquitously present in marine and agricultural habitats [25].

The present study aimed to investigate the potential of piericidin A to trigger a tauopathy *in vivo* in wild type mice or to modify the course of a genetically caused tauopathy in transgenic mice overexpressing human P301S mutant tau [26]. Therefore, we treated P301S tau transgenic mice and wild-type mice with Piericidin A or vehicle by subcutaneous infusion over a period of 28 days and analyzed their brains for the presence and severity of tau-pathology.

## Methods

### Animals

P301S transgenic mice were developed by Prof. Michel Goedert, Division of Neurobiology, University of Cambridge (Cambridge, UK). The detailed description of the animal model can be found elsewhere [26]. Briefly, the P301S tau mutation - position counted in the longest human isoform, with 441 amino acids (aa) - was cloned into the cDNA of the shortest four-repeat tau isoform (383 aa, in comparison to the 441 aa isoform, this isoform lacks in exons 2 and 3). This construct was then cloned into a murine thy1.2 expression vector at the XhoI site. After removal of the vector sequences, transgenic animals were generated by pronuclear microinjection of F1 embryos of mixed C57BL/6J × CBA/ca mice. Founder animals, identified by PCR analysis were intercrossed with C57BL/6J mice to establish lines [26]. Homozygous P301S^+/+^ and non-transgenic wild-type mice used for the study were kept in the same C57BL/6J background. All animals were 12 weeks of age at the beginning of the treatment period.

### Preparation of the minipumps

**Figure 1:**
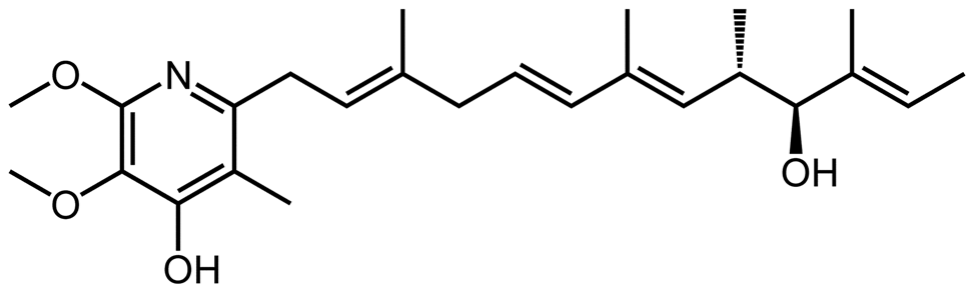
Chemical structure of piericidin A.

Piericidin A (Figure 1; Santa Cruz Biotechnology, Inc., Santa Cruz, CA, USA), was diluted in equal volumes of dimethyl sulfoxide (DMSO, Sigma-Aldrich, St. Louis, MO, USA) and polyethylene glycol 400 (PEG). Osmotic minipumps (ALZET model 2ML4, DURECT Corporation, ALZET Osmotic Pumps, Cupertino, CA, USA) were either filled with solutions containing piericidin A or vehicle (50% DMSO and 50% PEG) and were incubated in sterile NaCl (0.9% wt/vol) for 4 h prior to implantation.

### Surgery

The animal work was approved by the appropriate governmental authority (Regierungspräsidium Giessen, Germany), and conducted in accordance to the European Community Council Directive 86/609/EEC. For the experiments only male animals were used. In total, 19 transgenic animals (P301S^+/+^) and 16 wild-type animals were operated. The average weight of the animals was 28 g. For the surgical procedure, the animals were anaesthetized by intraperitoneal (i.p.) injection of ketamine (10 mg/kg) and xylazine (20 mg/kg) diluted in saline. Then a small skin cut was made in the neck region of the animals and anatomical forceps were used to open a subcutaneous pocket in the back of the animals. Osmotic minipumps were placed in this skin pocket and the skin was sutured.

### Infusion of piericidin A or vehicle

P301S^+/+^ and non-transgenic wild-type mice were exposed to piericidin A or vehicle for 28 consecutive days via permanent subcutaneous infusion. The infusion rate of piericidin A was 0.014 mg/d (equivalent to a daily dose of 0.5 mg/kg) in a volume of 7 µL buffer per day, summing up to a total amount of 0.392 mg piericidin A per animal, infused over the whole experimental period of 28 days. Vehicle treated animals were infused with the same volume of buffer solution as vehicle. Of the 19 P301S^+/+^ mice, 9 were exposed to piericidin A and 10 to the vehicle. Of the 16 wild-type animals, 9 were exposed to piericidin A and 7 to the vehicle. During the exposure period the animals were kept at a temperature of 21°C at a humidity of 55% under a 12 h/12 h light/dark cycle with free access to food and water.

### Tissue preparation

After the 28 days infusion period, the mice were deeply anaesthetized as described above and transcardially perfused with ice-cold 0.1 M phosphate buffered saline (PBS) for 2 minutes. Thereafter, the brains were quickly removed. The hemispheres were separated by a mid-sagittal cut. One hemisphere was post-fixed in 4% (wt/vol) paraformaldehyde in 0.1 M PBS for 24 h, cryoprotected in 10% (wt/vol) sucrose in 0.1 M PBS for 48 h, frozen in isopentane at −30°C for 2 min and stored at −80°C for histological analysis. The other hemisphere was immediately dissected, frozen in −30°C isopentane and stored at −80°C for Western blotting.

### Western blotting

Small pieces of frontal cortex where cut from the frozen brains and lysed using the T-PER protein extraction buffer (Thermo Scientific Pierce Protein Research Products, Rockford, IL, USA), supplemented with protease inhibitor (cOmplete Protease Inhibitor Cocktail, F. Hoffmann-La Roche Ltd., Basel, Switzerland) and phosphatase inhibitor (PhosSTOP Phosphatase Inhibitor Cocktail, F. Hoffmann-La Roche Ltd.), followed by centrifugation at 13,000 g for 15 min. Supernatants were loaded with Laemmli sample buffer (Bio-Rad, Laboratories, Hercules, CA, USA) to 12.5% SDS gels containing 10 µg protein per lane, separated by electrophoresis, and blotted to polyvinylidene difluoride membranes. These were blocked with 0.1 M tris buffered saline containing 0.1% (vol/vol) Tween (TBST, Sigma-Aldrich) and 5% (wt/vol) of skim milk powder (Sigma-Aldrich) for the HT7 blot or 10% (vol/vol) Roti-Block (Carl Roth, Karlsruhe, Germany) for the phospho tau blots for at least 1 h at room temperature and incubated overnight at 4°C with TBST, 5% (vol/vol) bovine serum albumin (BSA, Cell Signaling Technology, Inc., MA, USA), and the following primary antibodies: tau monoclonal antibody clone HT7 (MN1000, Thermo Scientific Pierce Protein Research Products; 1∶1000), AD2 anti-tau protein mouse monoclonal antibody against tau phosphorylated at serine 396 and serine 404 (mA #56484, Bio-Rad Laboratories, 1∶2000), mouse monoclonal phospho-PHF-tau pThr231 antibody (AT180) against tau phosphorylated at threonine 231 (MN1040, Thermo Scientific, Rockford, IL, USA, 1∶500), mouse monoclonal phospho-PHF-tau pSer202/Thr205 antibody (AT8) against tau phosphorylated at serine 202 and threonine 205 (MN1020, Thermo Scientific, 1∶1000), anti-phospho-PHF tau pThr212/Ser214 antibody (AT100) against tau phosphorylated at threonine 212 and serine 214 (MN1060, Thermo Scientific, 1∶1000). After three washes with TBST, the membranes were incubated for 2 h at room temperature with a horseradish peroxidase-conjugated secondary antibody (anti-mouse IgG, PI-2000, Vector Laboratories, Burlingame, CA, USA; 1∶3000). Protein loading was controlled for by reprobing the membranes with a chicken anti-glyceraldehyde-3-phosphate dehydrogenase (GAPDH) antibody (AB2302; EMD Millipore, Billerica, MA, USA; 1∶2000) and a horseradish peroxidase-conjugated secondary antibody (goat anti chicken IgY-HRP, sc-2428, Santa Cruz Biotechnology, Inc, 1∶5000). For visualization, membranes were incubated with Pierce ECL Western blotting substrate (Thermo Scientific Pierce Protein Research Products). Signals were detected using the ChemiDoc-XRS system (Bio-Rad Laboratories). For quantification all Western blot results were normalized to GAPDH as control protein.

### Immunohistochemistry

Brains were cut into 40 µm thick sections with a cryostat (Leica Biosystems, Wetzlar, Germany), collected in 10 regularly spaced series, and stored in 0.1 M phosphate buffer (PB) containing 0.01% (wt/vol) sodium azide at 4°C. Free floating sections were incubated subsequently for 15 min with 0.1% (vol/vol) H_2_O_2_ in 0.1 M PB to block endogenous peroxidase activity, for 1 h with 5% (vol/vol) normal donkey serum (NDS, Vector Laboratories) in 0.1 M PB and 0.2% Triton X-100 (Sigma-Aldrich) to inhibit non-specific binding sites, and for 24 h at 4°C with the following primary antibodies: AD2 anti-tau protein mouse monoclonal antibody (mA #56484, Bio-Rad Laboratories, 1∶1000), mouse monoclonal phospho-PHF-tau pThr231 antibody (AT180, MN1040, Thermo Scientific, 1∶1000), mouse monoclonal phospho-PHF-tau pSer202/Thr205 antibody (AT8, MN1020, Thermo Scientific, 1∶100), anti-phospho-PHF tau pThr212/Ser214 antibody (AT100, MN1060, Thermo Scientific, 1∶100), anti-NeuN mouse monoclonal antibody, clone A60 (MAB377, EMD Millipore, 1∶1000), anti-synaptophysin mouse monoclonal antibody, clone SY38 (MAB5258, EMD Millipore, 1∶2000). All primary antibodies were diluted in 0.1 M PBS with 5% (vol/vol) NDS and 0.2% (vol/vol) Triton X-100. After incubation with the primary antibody the sections were rinsed three times with 0.1 M PB. Sections were then incubated for 2 h at room temperature with the appropriate biotinylated secondary antibody (anti-mouse IgG, Jackson ImmunoResearch, West Grove, PA, USA, 1∶200) in 0.1 M PB with 5% NDS. The signal was amplified with the avidin–biotin method (VECTASTAIN Elite ABC Kit, Vector Laboratories, 1∶200). Bound antibodies were visualized with 0.5 mg/mL of 3,3′-diaminobenzidine tetrachloride (DAB, Sigma-Aldrich). A positive signal was obtained by oxidation of the dye by adding 1% H_2_O_2_ (vol/vol) in water which led to a brownish color. After 2 min of incubation with H_2_O_2_ the sections were rinsed three times with PB 0.1 M. To exclude non-specific labeling, sections were incubated as described above with the respective primary antibody omitted.

### Stereology

The cell numbers of phospho-tau (AD2, AT180, AT8, AT100) and NeuN-labeled neurons were estimated stereologically in the frontal cortex of one hemisphere by an observer blinded to the animals' identity, on regularly spaced (1/10) sections (average post-processing thickness 20 µm) under a 40× objective on an Olympus Microphot with the optical fractionator method, using the Stereo Investigator software (MicroBrightField, Inc., Williston, VT, USA). The frontal cortex was analyzed between 1.94 and 0.86 mm anterior and 0 to 2.0 mm lateral from the bregma. Coordinates were based on Paxinos and Franklin [27].

The criterion for counting a neuron was the presence of its nucleus either within the counting frame, or touching its right or top limit, but not touching its left or bottom limit. Total cell numbers were estimated by integration along the rostrocaudal extent of the structures. This sampling strategy gave a Schäfer coefficient of error ≤0.09.

### Optical density measurement

To quantify synaptic density, the immunoreactivity for the synaptic vesicle protein synaptophysin was measured. Therefore images of the whole section were taken with an Olympus-E330 camera with a Componon-S 2.8/50 magnification lens, attached to a light table (Copylizer eVision exe.cutive, Kaiser Fototechnik, Buchen, Germany). Color images were then converted to monochrome images and inverted using ImageJ software (National Institute of Health, USA). The frontal cortex and corpus callosum region were delineated on three consecutive sections per animal and the average grey values were measured. Because of the lack of synapses in the corpus callosum, the value obtained in this region was considered as background and subtracted of the values obtained in the frontal cortex to correct for non-specific background staining.

### Statistical analysis

Statistical analysis was done, using GraphPad Prism 6.0 (GraphPad Software, La Jolla, CA, USA). Results were expressed as mean ± SEM. The experimental groups were compared with a two-way analysis of variance (ANOVA) with the Fisher's LSD post-hoc test. A p-value <0.05 was assumed to be statistically significant.

## Results

### Low mortality due to experimental procedures

Altogether, 35 animals were used for this study. They were distributed in four groups: 10 P301S^+/+^ mice with vehicle treatment, 9 P301S^+/+^ mice with piericidin A treatment (0.5 mg/kg/d), 7 wild-type mice with vehicle treatment, and 9 wild-type mice with piericidin A treatment (0.5 mg/kg/d). All 35 animals survived anesthesia and the surgical procedure. In the group of wild-type mice treated with piericidin A, two died before the end of the 28 days infusion period and were excluded from further analysis. In the other three groups, all animals survived.

### Piericidin A exposure led to reduced cortical synaptic density in P301S^+/+^ mice, but not to cell death

To evaluate, whether piericidin A induced neurodegeneration, the total number of cells in the frontal cortex showing immunoreactivity against neuronal nuclei (NeuN) was counted stereologically. Since the reduction of synaptic density is considered to be an early sign of neurodegeneration [28], we also quantified the synaptic density in the frontal cortex by optical density measurement of immunoreactivity against the synaptic marker synaptophysin.

**Figure 2:**
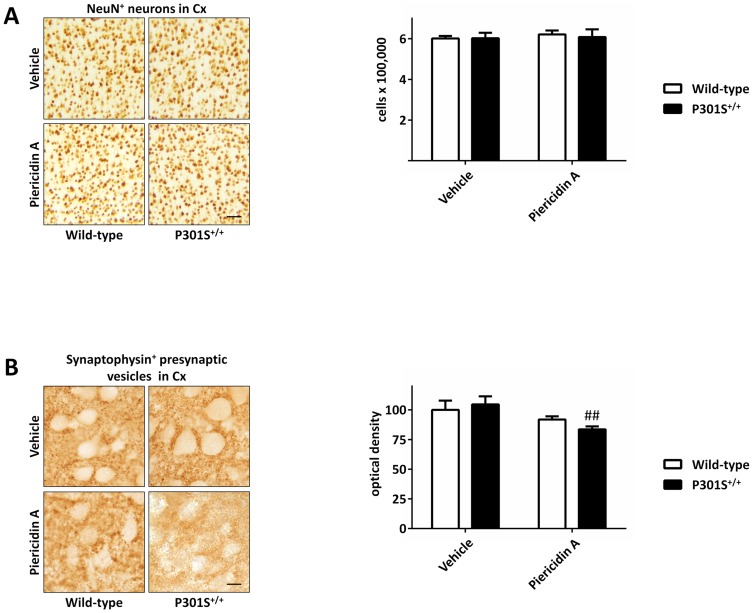
Effects of piericidin A treatment on neuronal cell count and synaptic density. A: Neuronal nuclei (NeuN)-stained sections of the frontal cortex (Cx) of wild-type and P301S^+/+^ mice treated with vehicle or piericidin A (left panel). Quantification of NeuN immunoreactive cells in the cortex showed no difference in the four animal groups (right panel). Scale bar 50 µm. B: Sections of the frontal cortex stained with an antibody against synaptophysin as marker for the synaptic density (left panel). The immunoreactivity quantified by optical density measurement was significantly reduced in piericidin A treated P301S^+/+^ mice compared to vehicle treated P301S^+/+^ (right panel). Scale bar: 10 µm. ## p <0.01, piericidin A treatment vs vehicle treatment at the same genotype. Two-way ANOVA with Fisher LSD post-hoc test.

After 28 days of treatment with piericidin A or vehicle, we did not observe any significant difference in the NeuN counts in the frontal cortex between the four groups (Figure 2 A). In wild-type mice there was also no significant difference in the synaptic density between vehicle treated (100±7.8%) and piericidin A treated mice (91.9±2.7%, Figure 2 B, empty bars). However, the synaptophysin immunoreactivity, likely to reflect synaptic density, was significantly reduced in piericidin A treated P301S^+/+^ mice (83.7±2.5% of vehicle treated non-transgenic mice) compared to vehicle treated P301S^+/+^ mice (104.5±6.8%, p = 0.008, Figure 2 B).

### Piericidin A exposure increased cortical tau-pathology in P301S^+/+^ mice, but not in wild-type mice

**Figure 3:**
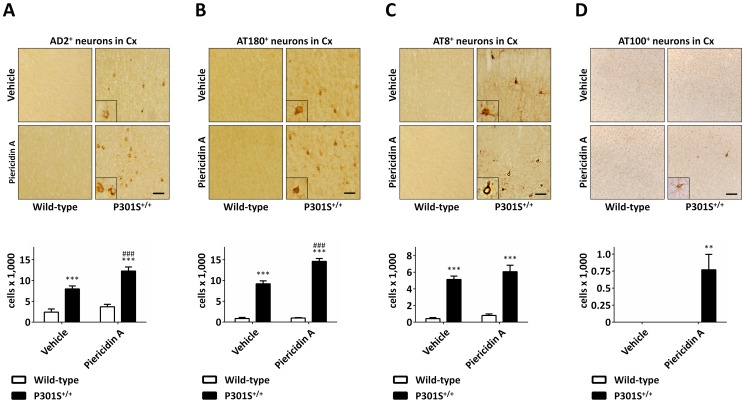
Histological sections showing the effect of piericidin A treatment on tau pathology. Frontal cortex (Cx) of wild-type and P301S^+/+^ mice treated with vehicle or piericidin A. Sections were stained separately with the antibodies AD2 (A), AT 180 (B), AT8 (C), and AT100 (D). The inserts are 2.5-times magnified compared to the overview images. P301S^+/+^ mice had significant higher numbers of phospho-tau positive cells in the frontal cortex compared to wild-type animals with the same treatment (lower panels, filled bars). Treatment with piericidin A further increased the number of AD2 immunoreactive cells (A, lower panel) and AT180 immunoreactive cells (B, lower panel). With AT8 immunoreactivity there was the same tendency, however, the difference was not significant (C, lower panel). With the AT100 antibody we only observed immunoreactive cells in piericidin A treated P301S^+/+^ mice (D). Scale bars: 20 µm. * p<0.05, ** p<0.01, *** p<0.001, P301S^+/+^ vs. wild-type at the same treatment. # p<0.05, ## p<0.01, ### p<0.001, piericidin A treatment vs. vehicle treatment at the same genotype. Two-way ANOVA with Fisher LSD post-hoc test.

To measure tau-pathology in the frontal cortex, brain sections were stained immunohistochemically with four different antibodies against tau, phosphorylated at different sites (AD2: pSer396/Ser404, AT8: pSer202/Thr205, AT180: pThr231, AT100: pThr212/Ser214). Immunoreactive cells were counted stereologically. After 28 days, vehicle treated wild-type mice showed only very little immunoreactivity against AD2, AT8, and AT180 and no immunoreactivity against AT100. Interestingly, treatment with piericidin A did not lead to a significant increase in the number of cells immunoreactive for AD2, AT8 or AT180 in wild-type animals (Figure 3 A, B, C, images on the left sides in the upper panels and white bars in the lower panels). In contrast, vehicle treated P301S^+/+^ mice already showed a considerably large number of cells with AD2, AT8 and AT180 immunoreactivity. (Figure 3 A, B, C, images on the right sides in the upper panels and black bars in the lower panels) However, in vehicle treated P301S^+/+^ mice, no AT100 immunoreactivity was detected (Figure 3 D). Treatment of P301S^+/+^ mice with piericidin A further increased the number of cells immunoreactive for AD2 (12,300±969 vs. 7,986±720, p = 0.0003) and AT180 (14,607±692 vs. 9,207±718, p<0.0001) compared to P301S^+/+^ mice treated with vehicle. (Figure 3 A, C, images on the right sides in the upper panels and black bars in the lower panels). With AT8 immunoreactivity, there was the same tendency, but the differences were not significant (p = 0.18, Figure 3 C, images on the right side in the upper panel and filled columns in the lower panel). Notably, piericidin A treated P301S^+/+^ mice showed a considerable number of AT100^+^ neurons (770±226), while vehicle treated P301S^+/+^ mice showed no AT100 immunoreactivity (p<0.01, Figure 3 D).

### Piericidin A exposure increased cortical levels of human tau in P301S^+/+^ mice

**Figure 4:**
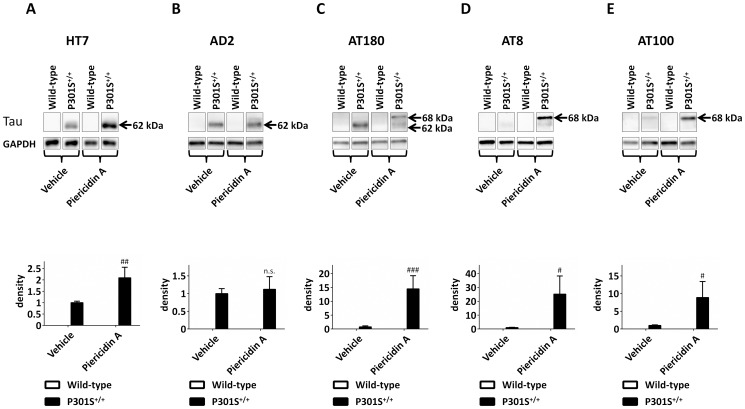
Western blots showing the effect of piericidin A treatment on total tau and phospho-tau levels. Upper panels: Western blots of wild-type and P301S^+/+^ mice, exposed to Piericidin A or vehicle over a period of 28 days, stained with an antibody specific for human tau (HT7, A), an antibody against pSer396/Ser404 (AD2) phosphorylated tau (B), an antibody against pSer202/Thr205 (AT8) phosphorylated tau, an antibody against pThr231 (AT180) phosphorylated tau (C), and an antibody against pThr212/Ser214 (AT100) phosphorylated tau (E). Below the tau Western blots the glyceraldehyde 3-phosphate dehydrogenase (GAPDH) loading control is shown. Lower panels: Quantification of the Western blots after normalization to loading control (GAPDH). In the HT7 and AD2 Western blots, the whole visible band was quantified, whereas in AT180, AT8 and AT100 the 68 kDa band was quantified. # p<0.05, ## p<0.01, ### p<0.001, n.s. not significant, piericidin A treatment vs. vehicle treatment at the same genotype. Two-way ANOVA with Fisher LSD post-hoc test.

To investigate whether treatment of P301S^+/+^ mice with piericidin A over a period of 28 days also led to increased levels of transgenic tau, we conducted Western blots analysis using an antibody specific for human tau (HT7). Therefore, we took tissue samples from eight animals of each group. We observed a 2.1±0.5-fold increase of human tau levels in P301S^+/+^ mice exposed to piericidin A compared to vehicle treated P301S^+/+^ mice (p = 0.01, Figure 4A).

### Piericidin A exposure led to increased cortical levels of pathologically phosphorylated tau in P301S^+/+^ mice

Wild-type animals did not show immunoreactivity for phosphorylated tau with any of the four tested phospho-tau antibodies in Western blot analysis (AD2, AT180, AT8, AT100), regardless of treatment with piericidin A or vehicle (Figure 4 A–E). In vehicle and piericidin treated P301S^+/+^ mice, AD2 and AT180 antibodies labeled a band of phopsphorylated tau at approximately 62 kDa (Figure 4 B and C). When staining with AD2, this band did not show significant alterations in intensity upon piericidin A treatment. With AT180, a decrease in intensity of this band was observed in piericidin A treated P301S^+/+^ mice compared to vehicle treated P301S^+/+^ mice (Figure 4 C). However, with AT180 we observed a second, higher molecular phospho-tau band at approximately 68 kDa, which was only detected in the piericidin A treated P301S^+/+^ mice (Figure 4 C). The total level of AT180-reactive phospho-tau did not show a significant increase after treatment with piericidin A. Interestingly, AT8 and AT100 antibodies only detected this higher molecular weight band at 68 kDa. AT8 labeling revealed this band exclusively in the piericidin A treated P301S^+/+^ mice (Figure 4 D). AT100 labeling revealed the 68 kDa band in both, piericidin A and vehicle treated P301S^+/+^ mice, but its intensity was significantly increased upon piericidin A treatment (Figure 4E).

To investigate whether increased tau levels per se were responsible for the increased levels of phosphorylated tau, we measured the ratio of phosphorylated tau to total human tau. There was no significant difference in the AD2/HT7 ratio between P301S^+/+^ mice treated with piericidin A or vehicle. There was 11.6±4.3 fold increase (p = 0.04) of AT180/HT7 in piericidin A treated P301S^+/+^ mice compared to vehicle treated P301S^+/+^ mice, when quantifying the 68 kDa phospho-tau bands. AT8/HT7 was also significantly increased by 4.2±1.1 (p = 0.008) and AT100/HT7 was increased by 10.1±5.3 (p = 0.12), ranging from 1.2-fold to 23.6-fold in piericidin A treated P301S^+/+^ mice, compared to vehicle treated P301S^+/+^ mice. Most likely due to this high range, the increase of AT100/HT7 did not reach significance, even though it was seen in all P301S^+/+^ mice upon piericidin A treatment. Altogether, these data suggest, that the observed increase of the levels of pathologically phosphorylated tau at the AT180, AT8, and AT100 epitopes in P301S^+/+^ mice treated with piericidin A compared to vehicle treated P301S^+/+^ mice was an effect independent of a mere increase of total human tau.

## Discussion

The aim of the present study was to investigate, if a synergistic interaction between a genetic predisposition and the exposure to an environmental factor may be relevant for the etiology of neurodegenerative tauopathies.

Therefore, we treated P301S tau transgenic mice and wild-type mice with piericidin A, an inhibitor of the mitochondrial complex I of microbial origin or vehicle, respectively, via subcutaneous pump infusion for a period of 28 days. Whereas we did not observe any neuronal cell loss, piericidin A led to a reduced immunoreactivity for synaptophysin and aggravated tau pathology in P301S^+/+^ mice, but not in wild-type mice.

Because the P301S^+/+^ mice develop typical histopathological features of tauopathies, which can also be found in post mortem tissue from patients [26], they are very suitable to study different aspects of the etiology and pathophysiology of neurodegenerative tauopathies.

Piericidin A, a very lipophilic inhibitor of mitochondrial complex I, was used, because the bacteria *(streptomyces spp.)* which synthesize piericidins, are living ubiquitously in normal soils, forest soils, or compost all over the world [25]. Therefore a contamination of fruits and vegetables with metabolic products of *streptomyces spp.* or the syntheses of these toxins within the human body appear to be possible ways of chronic exposure to bacterial toxins like piericidin A. Moreover, reports of *streptomyces spp.* causing diseases in patients show that a contamination of the human body with these bacteria is possible [29], [30].

In the present study, we did not observe cortical cell loss after a 28 days treatment period in the four animal groups. Since there were no data available about the bioavailability or efficacy of piericidin A *in vivo*, we chose the treatment doses on the basis of previous studies with lipophilic complex I inhibitors [19], [31], [22].

In two previous *in vivo* studies neurodegeneration was observed in rats after exposure to rotenone [31] or annonacin [19]. In the latter study annonacin was administered at doses of 3.8 mg/kg bodyweight or 7.6 mg/kg bodyweight of annonacine per day for a period of 28 days [19]. In a cell free assay, the potency of piericidin A to inhibit mitochondrial complex I was ∼2 fold smaller than the one of annonacin. In cultured neurons, the potency of piericidin A to induce the redistribution of phosphorylated tau from the dendrites into the cell soma and to induce cell death of piericidin A was ∼30-fold higher than that of annonacin [22]. In consideration of these data, a dose of 0.5 mg/kg body weight of piericidin A, which is ∼15-fold smaller than 7.6 mg/kg dose of annonacin, seemed to be an appropriate dose to investigate effects of piericidin A *in vivo*. Possible reasons, why we did not observe cell loss, could be the different mode of application (subcutaneously, not intravenously) as well as the different species (mice, not rats), used here in comparison to the previous rotenone and annonacin work. This subcutaneous way of application was chosen, because it appeared to be more physiological than an intravenous application. A further reason, why we did not observe cell loss, might be the relatively young age of the mice at the beginning of the treatment period. Allen et al. observed spinal cord neurodegeneration and a severe clinical phenotype with paraparesis and general weakness in these mice at an age of 6 months [26]. In the present study, we treated mice with an age of 12 weeks at the beginning of the treatment period. This age was chosen, because we wanted to observe effects that precede severe neurodegeneration. It is likely that young mice have more efficient compensatory mechanisms than old mice to prevent neurons from dying. Accordingly, in patients carrying a P301S mutation there was also no cell loss observed in early stages of the disease [32].

Even though there was no cell loss in the four animal groups, we observed a significant reduction of synaptophysin immunoreactivity in P301S^+/+^ mice that were treated with piericidin A. Because the immunoreactivity for this synaptic marker presumably reflects synaptic density, our observation corresponds to a reduction of synaptic density in P301S^+/+^ mice with piericidin A treatment. This can be considered as an early sign of neurodegeneration and was also observed by others in a different P301S tau transgenic mouse line [33] as well as in patients [34], where it is considered to contribute to cognitive impairment [35]. Neither the toxin alone nor the transgene alone had a significant influence on the cortical synaptic density. The fact, that piericidin A led to a reduced synaptic density only in P301S^+/+^ mice, suggests that there was a synergistic effect between the exposure to the toxin and the genotype of the mice.

As expected, we observed tau pathology in P301S^+/+^ mice. Both groups of tau transgenic animals, vehicle or piericidin A treated, showed a significant number of neurons with immunoreactivity for phosphorylated tau. Piericidin A treatment however, led to a marked increase in the number of phospho-tau positive neurons in P301S^+/+^ mice. Moreover, we only observed AT100^+^ neurons in piericidin A treated P301S^+/+^ mice, but not in vehicle treated P301S^+/+^ mice. In wild-type mice, on the other hand, neither the group of vehicle treated nor the group of piericidin A treated animals showed substantial signs of tau pathology. A similar observation was made in a recently published study, where mice, expressing human tau with the R406W mutation, which also belongs to the FTDP-17 mutations [36], were treated with annonacin subcutaneously for three days. There was also an increase in tau pathology only in the transgenic mice, while no tau pathology was observed in wild-type mice [37]. These observations further emphasize that there is a synergistic effect between the genotype and the exposure to an environmental toxin.

In accordance with the data from annonacin [37], piericidin A led to an increase in the levels of human tau in P301S^+/+^ mice, as confirmed by Western blot analysis in the present study. Moreover, piericidin A treatment led to increased levels of pathologically phosphorylated tau in P301S^+/+^ mice as shown by the formation of a higher molecular weight phospho-tau band at 68 kDa, which was either completely absent (AT8, AT180), or present only to a much lower extent (AT100) in the vehicle treated P301S^+/+^ mice. This shift to higher molecular weight phospho-tau bands was previously described and associated with pathological tau phosphorylation [38]. Moreover, a recent work showed, that bands with a lower molecular weight can appear as unspecific bands with some commercially available tau antibodies [39]. Thus, the higher molecular band we observed in piericidin A treated P301S^+/+^ mice might be the true correlate for pathologically phosphorylated tau, while the lower bands seen in AD2 and AT180 appearing at 62 kDa, might be non-specific.

The fact, that we observed a reduced synaptic density with piericidin A treatment in P301S^+/+^ mice as well as an increase in tau pathology after subcutaneous administration shows, that the compound was able to pass multiple biological membranes and penetrated to the brain of the mice. Likewise, it should be possible that piericidin A enters the brain after oral ingestion of contaminated food products. Admittedly, it is yet unknown, in what scale, fruits or vegetables are contaminated with bacterial toxins like piericidin A or the synthesizing bacteria themselves. However, because of the ubiquitary presence of these bacteria and the possibility that these very lipophilic toxins could accumulate in the brain over years, it seems worthwhile to further investigate, if the bacteria of their metabolites could be found in samples from patients suffering from a neurodegenerative tauopathy. This would provide further insight in the etiopathology of these diseases.

In summary, we showed for the first time, that piericidin A, a metabolic product of ubiquitously abundant bacteria, led to a loss of synaptophysin immunoreactivity and aggravation of tau pathology of P301S tau transgenic mice. We could show, that there was a synergistic interaction between the toxin and the genotype. Our results therefore provide further evidence that environmental toxins like piericidin A, rotenone or annonacin could be important factors in the etiology of tauopathies. This might be particularly the case in individuals at risk, having a specific genetic predisposition. Hence, avoiding the exposure to certain environmental toxins could be a way to prevent the development of neurodegenerative tauopathies or to slow the progress of these diseases.
