# Correction: Piericidin A Aggravates Tau Pathology in P301S Transgenic Mice

**DOI:** 10.1371/journal.pone.0122907

**Published:** 2015-03-26

**Authors:** The PLOS ONE Staff

There is an error in affiliation 7 for author Michel Goedert. Affiliation 7 should be: MRC Laboratory of Molecular Biology, Cambridge, United Kingdom.
